# Acute Toxicity and DNA Instability Induced by Exposure to Low Doses of Triclosan and Phthalate DEHP, and Their Combinations, *in vitro*

**DOI:** 10.3389/fgene.2021.649845

**Published:** 2021-04-20

**Authors:** Nathalia de Assis Aguilar Duarte, Lindiane Eloisa de Lima, Flora Troina Maraslis, Michael Kundi, Emilene Arusievicz Nunes, Gustavo Rafael Mazzaron Barcelos

**Affiliations:** ^1^Department of Biosciences, Institute of Health and Society, Federal University of São Paulo, Santos, Brazil; ^2^Institute of Environmental Health, Medical University of Vienna, Vienna, Austria

**Keywords:** co-exposure, endocrine-disrupting compounds, HepG2 cells, micronucleus, mutagenicity

## Abstract

Triclosan (TCS) is an antimicrobial agent widely used in personal care products (PCP) and the di-(2-ethyl hydroxy-phthalate) (DEHP) is a chemical compound derived from phthalic acid, used in medical devices and plastic products with polyvinyl chloride (PVCs). As result of their extensive use, TCS and DEHP have been found in the environment and previous studies demonstrated the association between their exposure and toxic effects, mostly in aquatic organisms, but there is a shortage in the literature concerning the exposure of TCS and DEHP in human cells. The aim of the present study was to assess the impact of exposure to TCS and DEHP, as well as their combinations, on biomarkers related to acute toxicity and DNA instability, in HepG2 cells, by use of cytokinesis-block micronucleus cytome (CBMNCyt) assay. For that, the cultures were exposed to TCS, DEHP and combinations at doses of 0.10, 1.0, and 10 μM for the period of 4 h and the parameters related to DNA damage (i.e., frequencies of micronuclei (MN) and nuclear buds (NBUDs), to cell division (i.e., nuclear division index (NDI) and nuclear division cytotoxic index (NDCI) and to cell death (apoptotic and necrotic cells) were scored. Clear mutagenic effects were seen in cells treated with TCS, DEHP at doses of 1.0 and 10 μM, but no combined effects were observed when the cells were exposed to the combinations of TCS + DEHP. On the other hand, the combination of the toxicants significantly increased the frequencies of apoptotic and necrotic cells, as well as induced alterations of biomarkers related to cell viability (NDI and NDCI), when compared to the groups treated only with TCS or DEHP. Taken together, the results showed that TCS and DEHP are also able to induce acute toxicity and DNA damage in human cells.

## Introduction

Triclosan (TCS) is a broad-spectrum antimicrobial compound used in personal care products (PCPs) (e.g., toothpaste, mouthwashes, soaps, deodorants), household cleaning products, toys, and plastics. TCS has been found frequently in rivers, effluents from sewage and water treatment plants, as well as in soil sediments (McAvoy et al., 2002; Agüera et al., 2003; Olaniyan et al., 2016). It is also known that the degradation of TCS may generate compounds of higher toxicity and greater persistence, such as dioxins and chlorophenols (Pusceddu et al., 2018).

Previous *in vitro* and *in vivo* studies showed that TCS presents estrogenic, antiestrogenic, and antiandrogenic properties, which appears to be related to cell lines, tissues, and animal species (for a comprehensive review, see Ishibashi et al., 2004; Kumar et al., 2009; Jung et al., 2012; Alfhili and Lee, 2019).

One of the most important mechanisms related to TCS-induced toxicity is the capacity to cause disturbances of the redox status of cells, leading to oxidative damage in lipids, proteins, and DNA (Binelli et al., 2009; Park et al., 2016; Zhang et al., 2018). Previous *in vivo* studies showed that TCS is able to induce genotoxicity in several aquatic organisms, such as algae, ciliated protozoa, crustaceans and microcrustaceans, and zebra mussels (Ciniglia et al., 2005; Binelli et al., 2009; Gao et al., 2015; Silva et al., 2015; Xu et al., 2015). On the other hand, few studies were carried out aiming to assess the impact of TCS exposure on mammalian organisms, either *in vitro* and *in vivo*. For example, Li et al. (2018) showed that exposure to TCS (concentrations ranging from 5.0 to 40 μM) was able to induce cell cycle arrest and apoptosis, in HepG2 cells. In weanling rats, Riad et al. (2018) found that TCS caused testicular DNA damage, increase of malondialdehyde (MDA), and decrease of superoxide dismutase (SOD). In humans, exposure to TCS was associated with high levels of urinary 8-hydroxy-2′-deoxyguanosine (8-OHdG) in children from China (Lv et al., 2016) and from Brazil (Rocha et al., 2018).

As well as TCS, the di-2-ethylhexyl phthalate (DEHP), derived from phthalic acid, is classified as endocrine-disrupting compounds (EDCs), which comprise a group of toxicants that interacts with natural hormones, interfering with their synthesis, secretion, binding, transport, and elimination, by changing their functions (Kabir et al., 2015). EDCs are widely present in products used in daily life, such as PCP, pesticides, food packaging, plastics, and flame retardants (Arambula and Patisaul, 2018). Due to its capacity to give malleability in polyvinyl chloride (PVCs), DEHP is widely used by several industries sectors, being found in medical devices, plastic packages, toys, building materials, among others (Rusyn et al., 2006; Caldwell, 2012).

DEHP is highly lipophilic and easily absorbed by the gastrointestinal tract, as well as by skin and lungs. Once absorbed, DEHP is quickly metabolized mainly in liver, resulting in several metabolites, such as the mono(2-ethylhexyl) phthalate (MEHP), which is associated with most of the toxic effects induced by DEHP exposure (Fay et al., 1999; Rusyn et al., 2006; Caldwell, 2012). Previous *in vitro* and *in vivo* studies also showed that DEHP can induce disturbances of cell homeostasis, leading to DNA instability, dysfunctions of the mitotic spindle, and cell death (Turner et al., 1974; Choi et al., 2010; Caldwell, 2012; Li et al., 2014; Rowdhwal and Chen, 2018; Silano et al., 2019).

HepG2 cells are widely used for testing the genotoxic properties of several environmental toxicants, mainly by use of micronucleus (MN) and of comet assays (for review, see Guo et al., 2020). The employment of this cell line is particularly advantageous due its ability to express several phases I and II drug metabolizing enzymes, which can catalyze the activation and detoxification of several toxicants, such as cytochrome P450 isoenzymes (CYP1A1, 1A2, 2B, 2C, 3A, and 2E1) and sulfotransferases (SULTs), glutathione-S-transferase (GSTs), UDP-glucuronosyltransferase (UGT), and N-acetyl-transferase (NAT); moreover, HepG2 cells also have proficient p53 gene expression, as well as functional DNA repair systems. Previous studies showed that p53-competent cells, with active expression of phases I and II drug metabolizing enzymes, as well as functional DNA repair systems may significantly reduce the rate of false positive results comprising genotoxicity assays (for review see Knasmüller et al., 2004; Mersch-Sundermann et al., 2004; Fowler et al., 2012; OECD, 2014).

As mentioned above, both chemicals are widely used on daily life and there is enough data comprising their hazard to aquatic organisms; however, it still has a lack of information of their behavior in mammalian cells. Both compounds are lipophilic, and it seems to cause damage after phase I drug metabolization system, resulting in compounds with higher toxicity (for a comprehensive review, see Caldwell, 2012; Wu et al., 2019); moreover, previous human biomonitoring studies have detected the coexposure of TCS and DEHP (or their metabolites), in urine samples of children and adults, in several regions worldwide (Casas et al., 2011; Larsson et al., 2014; Braun et al., 2017; Rocha et al., 2017, 2018; Lim, 2020; Li et al., 2021). Therefore, assessing to possible combined effect of TCS and DEHP would provide further information about molecular effects of these toxicants, as well as their interactions, in the environment and/or in organisms (Arambula and Patisaul, 2018). This study aimed to assess the impact of the exposure to TCS and DEHP, as well as their combination on biomarkers of acute toxicity and of DNA stability, in HepG2 cells.

## Materials and Methods

### Reagents

TCS (triclosan; 5-chloro-2-(2,4-dichlorophenoxy) phenol; CAS 3380-34-5), DEHP (bis(2-Ethylhexyl) phthalate; CAS 117-81-7), 3,4-benzopyrene (B[a]P; CAS 50-32-8), methyl methanesulfonate (MMS; CAS 66-27-3), cytochalasin B (Cyt-B; CAS 14930-96-2), dimethyl sulfoxide (DMSO; CAS 67-68-5), Giemsa (CAS 51811-82-6) and Dulbecco’s Modified Eagle’s Medium (DMEM, low glucose) were obtained from Sigma-Aldrich (St. Louis, MO, United States), Trypsin-EDTA (0.25%) and antibiotic-antimycotic solution (10,000 units/mL of Penicillin, 10,000 μg/mL of Streptomycin, and 25 μg/mL of Amphotericin B) came from Gibco (Grand Island, NE, United States) and fetal bovine serum (FBS) was purchased from LGC Biotecnologia (Cotia, Brazil). All other chemicals, reagents, and buffers were analytical grade products from Sigma-Aldrich (St. Louis, MO, United States).

### Cell Culture Conditions and Treatments

HepG2 cells were kindly provided by Prof. Dra. Lusânia M. G. Antunes from School of Pharmaceutical Sciences of Ribeirão Preto, University of São Paulo, Brazil.

Briefly, the cells were maintained in DMEM low glucose with 10% of FBS and 1.0% of antibiotic-antimycotic, in a CO_2_ incubator with 5% atmosphere at 37°C and 96% of relative humidity. All experiments were conducted between the third and eighth cell passage.

**FIGURE 1:**
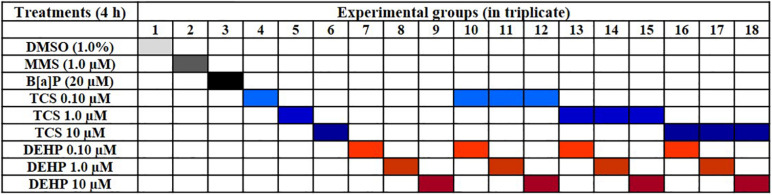
Schematic overview of treatments carried out in the study.

10^6^ cells were seeded in 25 cm^2^ cultures flasks for 24 h in the complete medium; after, the medium was removed, cells were washed twice with PBS (pH 7.2) and, then, cells were incubated with culture medium without FBS for the period of 4 h, with TCS, DEHP, as well as their combinations (Figure 1); moreover, vehicle (DMSO 1.0%) and positive controls (B[a]P 20 μM, and MMS 1.0 μM) were also included in the experiments. Concentrations of positive controls, of TCS, DEHP and their combinations were chosen based on previous MTT assays (data not shown).

### Cytokinesis-Block Micronucleus Cytome (CBMNCyt) Assay

CBMNcyt assay was carried out in three independent experimental replicates following the protocol published by Fenech (2007). After exposure of the cells to TCS, DEHP, and their combinations, cells were washed twice with PBS (pH 7.2) and incubated with cytochalasin B (3.0 μg/mL) in complete medium for further 30 h. After, cells were washed, trypsinized, and centrifuged for 5 min at 180 *g*. Then, the pellets were resuspended in 5.0 mL of hypotonic solution (sodium citrate 1.0%; 100 μL of formaldehyde (25%) at 4°C) for 4 min. The cells were centrifuged twice and fixed in with methanol and acetic acid (3:1 v/v) and then, slides were prepared. Slides were stained with 5.0% Giemsa in PBS (pH 7.2) and cells were analyzed by light microscopy (Carl Zeiss, AxioLab A1, Jena, Germany) at magnification of 630x.

In total, 3,000 binucleated cells (1,000 cells per experimental replicate) were analyzed for each experimental point. Analyses of nuclear anomalies named micronucleus (MN), nuclear buds (NBUDs), nucleoplasmatic bridges (NPBs), as well as the number of apoptotic and necrotic cells were carried out according to the scoring criteria described by Fenech (2007). Pictures of each assessed endpoint are depicted in Supplementary Figure 1. Moreover, nuclear division index (NDI) was scored in 1,500 cells (500 cells per experimental replicate) according to the formula proposed by Eastmond and Tucker (1989), as follow: *NDI* = *(M1* + *2.M2* + *3.M3* + *4.M4)/N*, where “M1-M4” represent the number of cells with one, two, three, and four nuclei, respectively, and “N” is the number of cells scored. Nuclear division cytotoxicity index (NDCI) was also scored in 1,500 cells (500 cells per experimental replicate) according to the formula proposed by Fenech (2000): *NDCI* = *(Ap* + *Nec* + *M1* + *2.M2* + *3.M3* + *4.M4)/N*, where “ap” and “nec” are the number of apoptotic and necrotic cells, “M1-M4” represent the number of cells with one, two, three, and four nuclei, respectively, and “N” is the number of cells scored (both viable and non-viable ones).

### Statistical Analyses

Nuclear anomalies (MN, NBUDs, and NPBs) and counts of necrotic and apoptotic cells were analyzed by Poisson regression. Overdispersion was tested by chi-square tests. Values of NDI and NDCI were log-transformed due to their skewed distribution and analyzed by a Generalized Linear Model with Gaussian deviates. Normality of residuals was tested by Kolmogorov-Smirnov tests with Lilliefors’ corrected *p*-values. Homogeneity of variances was assessed by Brown-Forsythe tests.

Values of exposed cells were compared to their controls by Wald’s chi-square tests with Bonferroni corrected *p*-values. Furthermore, cells treated with the combined exposures were compared to the group that was treated with its respective single exposure (i.e., TCS vs. TCS + DEHP; and DEHP vs. TCS + DEHP; at the same doses) using the same procedure. All analyses were done by Stata 13.1 (StataCorp, College Station, TX, United States).

## Results

Table 1 summarizes the impact of treatments of HepG2 cells with TCS, DEHP, as well as their combinations on the parameters of cell death, cell viability, and DNA damage.

**TABLE 1:** Impact of exposure to triclosan (TCS), to phtalate DEHP, as well as their combinations on biomarkers of cell death (apoptosis and necrosis), cell viability (nuclear division index, (NDI); and nuclear division citotoxicity index (NDCI) and DNA instability (micronuclei (MN), nuclear buds (NBUDs) and nucleoplasmatic bridges (NPBs), in HepG2 cells.

Treatments (μM)	Cell death		Cell viability		Mutagenic effects		
	Apoptosis	Necrosis	NDI	NCDI	MN	NBUDs	NPBs
**DMSO^*a*^ (1.0%)**	4.0 ± 1.1	5.0 ± 1.1	1.9 ± 0.040	1.8 ± 0.030	2.7 ± 1.1	1.0 ± 1.7	0
**MMS^*b*^ 1.0**	10 ± 2.3**	58 ± 3.2**	1.7 ± 0.11**	1.5 ± 0.020**	7.3 ± 3.1*	1.3 ± 1.1	0
**B[a]P^*c*^ 20**	4.0 ± 1.5	6.0 ± 3.6	2.0 ± 0.13*	1.8 ± 0.030	8.0 ± 0**	2.7 ± 3.1	0
**TCS 0.10**	13 ± 0.8**	6.0 ± 2.5	1.7 ± 0.040**	1.7 ± 0.040*	9.7 ± 1.5**	2.3 ± 2.3	0.33 ± 0.58
**TCS 1.0**	18 ± 6.3**	77 ± 7.0**	1.7 ± 0.010**	1.2 ± 0.020**	11 ± 2.5**	11 ± 6.8**	1.67 ± 0.58
**TCS 10**	4.0 ± 4.7	7.0 ± 0.82	1.8 ± 0.020	1.7 ± 0.020**	10 ± 5.0**	8.7 ± 8.1**	0.50 ± 0.71
**DEHP 0.10**	11 ± 2.5**	9.0 ± 1.1*	1.7 ± 0.090**	1.7 ± 0.030	3.3 ± 1.5	3.7 ± 1.5*	0
**DEHP 1.0**	30 ± 0.50**	16 ± 7.2**	1.9 ± 0.080	1.8 ± 0.10	14 ± 1.1**	14 ± 10**	1.0 ± 1.0
**DEHP 10**	13 ± 1.2**	33 ± 6.2**	1.9 ± 0.020	1.8 ± 0.050	17 ± 3.6**	13 ± 5.0**	1.7 ± 2.0
**TCS 0.10 + DEHP 0.10**	11 ± 3.3**	10 ± 0.90*	1.6 ± 0.10**	1.5 ± 0.10**	9.3 ± 1.5**	4.7 ± 2.1*	2.3 ± 1.5
**TCS 0.10 + DEHP 1.0**	23 ± 8.8**	20 ± 13**	1.6 ± 0.040**	1.5 ± 0.060**	6.3 ± 2.3*	1.7 ± 1.1	0
**TCS 0.10 + DEHP 10**	30 ± 11**	30 ± 24**	1.7 ± 0.090**	1.6 ± 0.16**	1.0 ± 0.0	0.33 ± 0.58	0
**TCS 1.0 + DEHP 0.10**	31 ± 23**	28 ± 20**	1.7 ± 0.050**	1.5 ± 0.17**	3.3 ± 0.58	15 ± 2.6*	0.33 ± 0.58
**TCS 1.0 + DEHP 1.0**	39 ± 20**	33 ± 20**	1.8 ± 0.10*	1.6 ± 0.11**	15 ± 2.6**	7.7 ± 4.5**	1.3 ± 1.5
**TCS 1.0 + DEHP 10**	2.0 ± 0.90	7.0 ± 1.7	1.8 ± 0.070**	1.8 ± 0.070	16 ± 2.0**	12 ± 8.0**	0.67 ± 0.58
**TCS 10 + DEHP 0.10**	9.0 ± 0.80*	11 ± 3.3*	1.7 ± 0.10**	1.7 ± 0.090**	6.0 ± 2.6	1.3 ± 0.58	0.33 ± 0.58
**TCS 10 + DEHP 1.0**	11 ± 7.3**	34 ± 3.7**	1.8 ± 0.070*	1.7 ± 0.13*	9.3 ± 4.9**	4.3 ± 3.2*	1.0 ± 1.0
**TCS 10 + DEHP 10**	38 ± 9.5**	54 ± 1.2**	1.7 ± 0.10**	1.6 ± 0.050**	11 ± 4.6**	8.7 ± 8.3**	0.50 ± 1.71

*^*a*^Negative (solvent) control: dimethyl sulfoxide. ^*b*^Positive control: methylmethane sulfonate (no required biotransformation/activation). ^*c*^Positive control: benzo[a]pyrene (required biotransformation/activation).* *p < 0.050; **p < 0.010 (both in bold) compared to vehicle control group (DMSO).

It can be seen that the lower and the intermediate doses of TCS (0.10 and 1.0 μM) were able to increase the number of cells in apoptosis when compared to the vehicle control group, while only the intermediate concentration of TCS (1.0 μM) increased significantly the number of necrotic cells; on the other hand, significant increase of apoptosis and necrosis were seen in the cells treated with all doses of DEHP (0.10–10 μM). Cotreatments of the cells with the combinations of TCS + DEHP increased significantly the percentage of both biomarkers related to cell death, with the exception of the treatment of TCS 1.0 μM + DEHP 10 μM. Concerning the parameters related to cell division kinetics, TCS presented higher cytostatic effects, when compared to the groups that were exposed to DEHP, measured by NDI and NDCI. Moreover, only the combination of TCS 1.0 μM + DEHP 10 μM did not induce significantly cytostatic effects, when compared to the respective vehicle control groups.

Concerning the parameters related to DNA instability, TCS, DEHP, and their combinations were able to increase the DNA damage of cells, assessed by MN and NBUDs endpoints. TCS at all concentrations (0.10, 1.0, and 10 μM) was able to increase the MN frequencies when compared to the vehicle control group and only the lowest dose of DEHP (0.10 μM) did not induce MN formation. When the HepG2 cells were exposed to the combination of TCS and DEHP, the treatments TCS 0.10 + DEHP 10; TCS 1.0 + DEHP 0.10, and TCS 10 + DEHP 0.10 did not increase the MN formations, when compared to the respective vehicle control group. It is also important to mention that a significant increase of MNs was seen in cells treated with MMS and B[a]P.

Increase of NBUDs formation was seen in the groups treated alone with TCS at the intermediate and highest concentrations, i.e., at 1.0 and 10 μM, while higher NBUDs frequencies were seen in the cells that were treated with all doses of DEHP (0.10–10 μM). Concerning the cells that receive the cotreatments with to TCS + DEHP, combinations between TCS 0.10 + DEHP 1.0, TCS 0.10 + DEHP 10 and TCS 10 + DEHP 0.10 did not increase significantly the formation of NBUDs. On the other hand, TCS, DEHP and their associations did not increase the frequencies of NPBs, when compared to the respective vehicle control.

**FIGURE 2:**
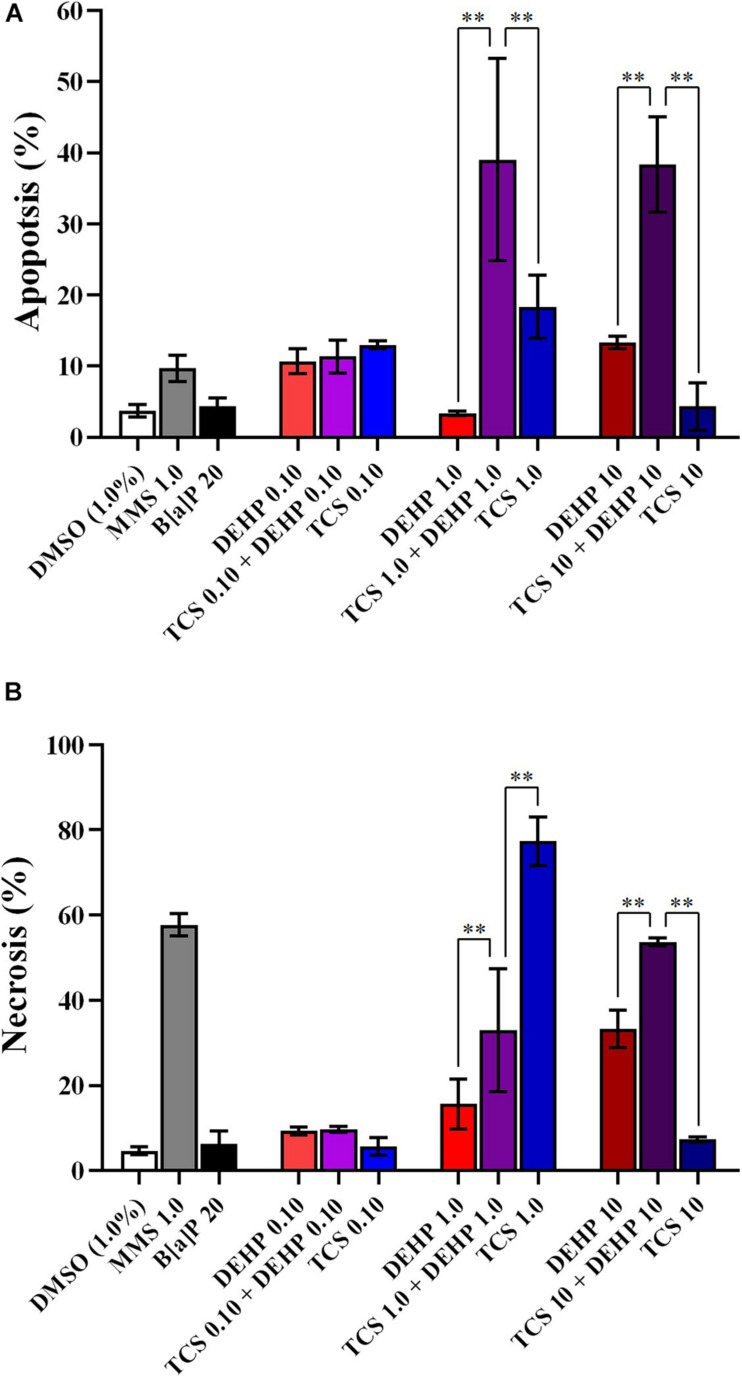
Comparisons between the treatments with TCS + DEHP and their respective groups that receive TCS or DEHP alone, on parameters of cell death: **(A)** apoptosis and **(B)** necrosis. All doses are in μM. ^∗∗^*p* < 0.010; Wald’s chi-square tests followed by Bonferroni correction tests.

**FIGURE 3:**
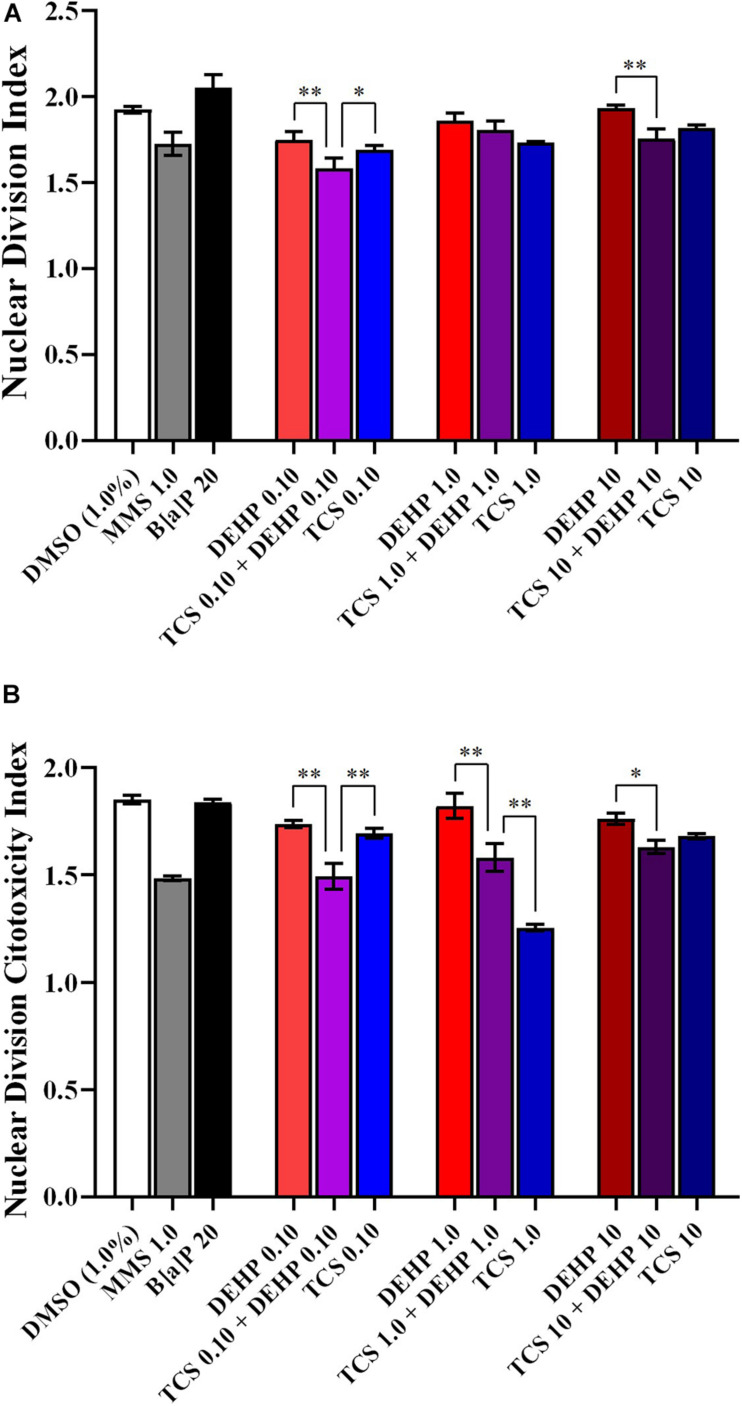
Comparisons between the treatments with TCS + DEHP and their respective groups that receive TCS or DEHP alone, on parameters of cell viability: **(A)** nuclear division index (NDI) and **(B)** nuclear division cytotoxicity index (NDCI). All doses are in μM. ^∗^*p* < 0.050; ^∗∗^*p* < 0.010; Wald’s chi-square tests followed by Bonferroni correction tests.

**FIGURE 4:**
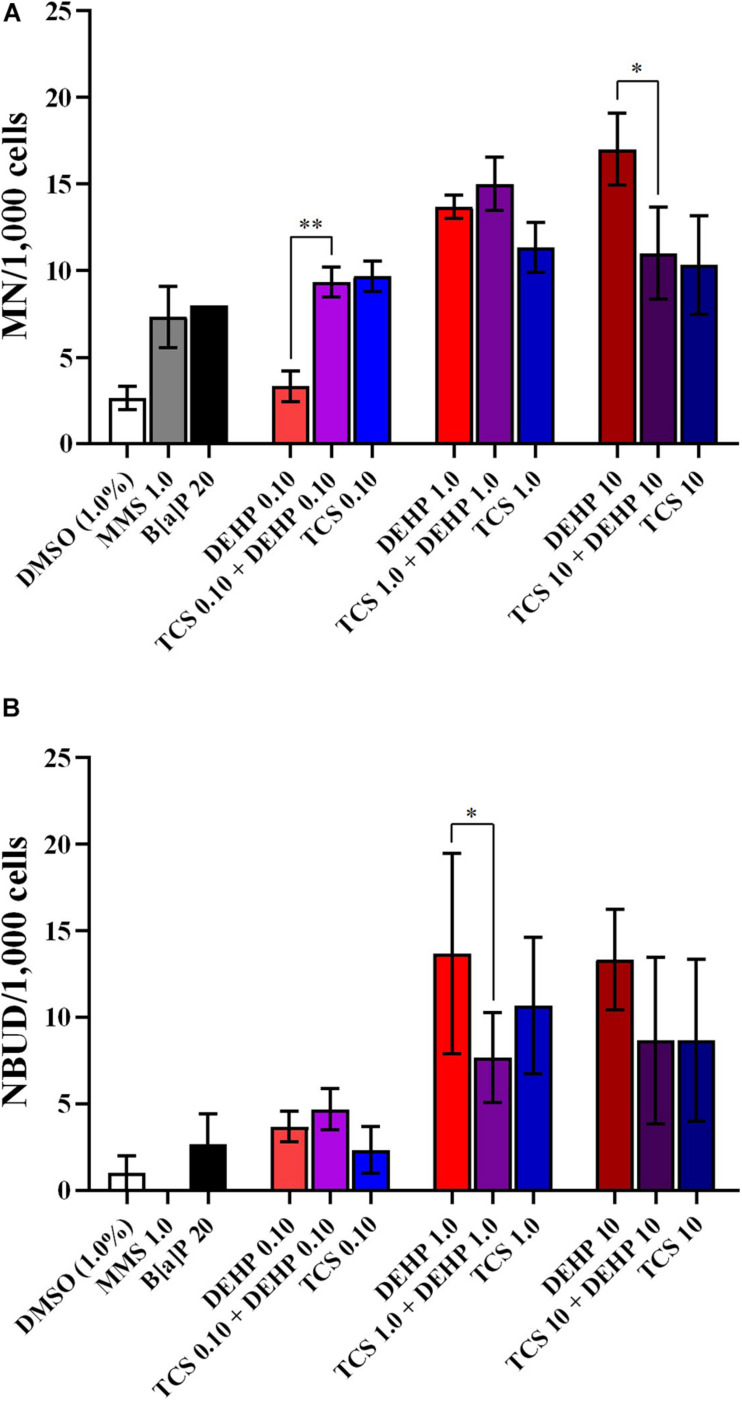
Comparisons between the treatments with TCS + DEHP and their respective groups that receive TCS or DEHP alone, on parameters of DNA damage: **(A)** micronuclei (MN) formation and **(B)** nuclear buds (NBUDs) formation. All doses are in μM. ^∗^*p* < 0.050; ^∗∗^*p* < 0.010; Wald’s chi-square tests followed by Bonferroni correction tests.

Figures 2–4 depict the comparisons between the treatments with TCS + DEHP and their respective groups that receive TCS or DEHP alone, on parameters of cell death (apoptosis and necrosis), of cell viability (NDI and NDCI), and DNA damage (MNs, NBUDs, and NPBs), respectively.

There were no significant differences in the percentage of apoptosis and necrosis among the cells that receive the lowest dose of TCS, DEHP, as well as their combination, while an increasing number of apoptotic cells were seen in the groups that were treated with TCS 1.0 + DEHP 1.0; and TCS 10 + DEHP 10, when compared to the cells that were exposed only to TCS and to DEHP at 1.0 and 10 μM, respectively. Cells treated with DEHP at 1.0 μM had lower necrotic events than the cotreatment with TCS + DEHP (both at 1.0 μM); on the other hand, treatment with TCS 1.0 μM increases the percentage of necrosis, when compared to the group that receives TCS 1.0 + DEHP 1.0 μM. It can be also observed a significant increase of necrotic cells in the treatment with the combination of TCS and DEHP (both at 10 μM) when compared to the groups that were exposed to the compounds alone (Figure 2).

Figure 3 illustrates the NDI and NDCI of cells treated with TCS, DEHP, and their associations. Combined effects were observed in the groups that receive the lowest doses of TCS and DEHP, i.e., cells treated with TCS + DEHP (both at 0.10 μM) showed a decrease of NDI and NDCI when compared to those that were exposed only to TCS or DEHP alone. Lower NDI and NDCI were also seen in the groups treated with TCS + DEHP (both 10 μM) when compared to the cells that receive only DEHP 10 μM, while no statistical difference was seen between TCS 10 μM and TCS 10 + DEHP 10. No differences of NDI were seen among groups that receive only TCS 1.0 μM; DEHP 1.0 μM and their association, while the lowest NDCI was observed in the cells treated with TCS 1.0 μM (DEHP 1.0 > TCS 1.0 + DEHP 1.0 > TCS 1.0).

Although the exposure to TCS and DEHP, as well as their combination, induces DNA instability, no combined effects were seen on the biomarkers related to DNA damage (MN and NBUD formations) (Figure 4); moreover, since none of assessed treatments induced NPBs formation, no combined effects can be seen for this endpoint (data not shown).

## Discussion

Previous studies showed that TCS exposure induces acute toxicity, leading to cell death. For example, Li et al. (2018) showed that TCS at concentrations of 5.0 and 10 μM was able to induce DNA instability, promoting apoptosis mediated by p53 expression, in HepG2 cells. Li et al. (2019) observed that PC12 cells exposed to TCS at doses of 10 and 50 μM for 12 h was able to activate the expression of p38 mitogen-activated protein kinase and Bax, which are related to apoptosis signaling. Moreover, Park et al. (2016) also showed that TCS-induced apoptosis in rat neural stem cells (NSCs) was mediated by Bax expression and activation of caspase 3, at the dose of 50 μM. Our findings showed that the concentrations of TCS at 0.10 and 1.0 μM increased the number of apoptotic cells, while the concentration of 1.0 μM enhanced the necrotic cells. Furthermore, Yueh et al. (2014) observed an increase of expression of the inflammatory cytokines named TNFα, TNFβ, and IL-6 in the liver of male mice fed with a diet containing 0.08% of TCS for 8 months. It is well established that the expression of TNFα, TNFβ, and IL-6 are associated with cell death signaling pathways, mainly by necrosis (D’Arcy, 2019).

Regarding to DEHP, Fang et al. (2019) assessed the impact of DEHP exposure at high dose (512 μM for 4 h) on parameters of cell viability, in differentiated human embryonic stem cells, and observed that exposure to the compound was able to induce apoptosis by triggering the PPARγ/PTEN/AKT pathway, which is related to cell proliferation and survival. Like to TCS, cell death induced by DEHP also appears to be related to Bax expression, as well as to an increase of caspases 3 and 8, as seen previously by and Hannon et al. (2016) and Sun et al. (2018), using *in vitro* laboratory models. Ha et al. (2016) observed an increase of apoptotic events, in hepatocytes of Sprague-Dawley rats treated with DEHP at 250, 500, and 750 mg/kg for 30 days; according to the authors, the increase of cell death was mediated by p53 overexpression caused by DEHP exposure. Our results demonstrated that DEHP can induce apoptosis even at lower concentrations, since we observed increases in cell death at doses of 0.10 and 1.0 μM.

We also observed that exposure to the both EDCs was able to impact the NDI of HepG2 cells and previous studies showed that oxidative damage in DNA is related to cell cycle arrest (Droge, 2002; Murray and Carr, 2018). Reduced NDI is associated with more mononucleated cells, when compared to bi-, tri-, and polynucleated ones, giving evidence of cell cycle arrest (Fenech, 2007, 2020). Interestingly, the most pronounced effects on NDI were seen when the cells were treated with the combinations of TCS and DEHP, at all doses. This parameter is a measure of the proliferative status of viable cells fraction, if the NDI is lower than control, it can be assumed that more cells with one nucleus were scored, suggesting cytostatic effects (Fenech et al., 2011). Therefore, one hypothesis for our observations may be related to the activation of DNA repair pathways. In the cell cycle, G2/M checkpoint prevents the entry of mitosis, when DNA damage was not properly repaired. Delaying entry into mitosis allows repair mechanisms of DNA lesions before cell division, avoiding passing the damage to the new cells (Li and Zou, 2005; Gomez and Hergovich, 2016; Choi and Chung, 2020). In the NDCI, necrotic and apoptotic cells are included in the number of cells scored, since the toxic effects induced by chemical compounds may provide a large proportion of cells becoming non-viable. Therefore, an overestimated cytostatic effect can be observed in NDI if necrotic and apoptotic cells were not included in the scoring (Fenech, 2000).

In our study, clear mutagenic effects were seen in the cells exposed to TCS, DEHP and their combinations, by significant increase of MN and NBUD frequencies. An earlier study carried by Li et al. (2019) suggested exposure to TCS may be related to double strand breaks (DSBs), since the authors observed a significant increase of comet formations in HepG2 cells treated with TCS at 20 and 40 μM, when compared to the negative control group; in the same investigation, the authors described that DNA-dependent protein kinase (DNA-PKcs) is required for the DNA double-strand break repair through the non-homologous end-joining (NHEJ) pathway. The formation of MN occurs during cell division when the genetic material is exposed to mutagenic compounds promoting the break of chromosomes (by clastogenic agents, which are related to acentric fragments) or loss of whole chromosomes (by aneugenic agents, which are related to disturbances of mitotic spindle formation); therefore, both (whole chromosomes or their fragments) are not incorporated into the main nucleus during the telophase and they are surrounded by a nuclear membrane, generating a MN (Fenech, 2007; Fenech et al., 2011). Earlier laboratory studies also described an association between DEHP exposure and increased DNA instability. For example, Kim et al. (2019) showed higher γH2AX formation in 8505C thyroid gland carcinoma cells treated with DEHP at concentrations ranging from 5.0 to 50 μM, while increased comet formations were seen in the cells treated with DEHP at doses from 5.0 to 100 μM. γH2AX is a very sensitive biomarker associated with cellular response to the induction of DNA double-strand breaks (DSBs) (Kopp and Audebert, 2019).

Our results provide further pieces of evidence that TCS and DEHP are able to induce chromosome breaks, which resulted in an increase of MN frequencies, especially when the cells were exposed simultaneously to both chemicals. In addition, NBUDs have a similar morphology to MN; however, they are connected to the main nucleus by a stem of nucleoplasmatic material. Most of the NBUDs originate from interstitial or terminal acentric fragments (Fenech, 2007; Fenech et al., 2011). In this context, MNs may also arise through gene amplification through fusion bridge breaking (BFB) cycles. In the case of NBUDs, DNA is selectively located at specific locations on the periphery of the nucleus and can be eliminated through nuclear sprouting, during S phase of the cell cycle; then, as consequence, extrusion of amplified DNA via nuclear sprouting may result in MN formations (Fenech and Crott, 2002; Mateuca et al., 2006; Fenech, 2020). It is also important to mention that we did not observed increase of NPBs in cells treated with TCS and DEHP. According to Bonassi and Fenech (2019), NPBs breaks generate a pair of abnormal chromosomes lacking telomeres and results in further end-fusion and gene amplification; amplified gene sequences and unresolved DNA repair complexes are removed by NBUDs formations.

Previous data report that toxicant mixtures may increase the adverse effects when compared to one only exposure (Kabir et al., 2015; Hernández et al., 2017). On the other hand, the chemical compounds may compete for similar metabolizing pathways, resulting in high biotransformation rating, which can increase or decrease the toxic effects of xenobiotics (Lin, 2006). These interactions depend on several factors, including affinity and concentration of substrates, time of exposure, and CYP450 system inhibition, for example (Deodhar et al., 2020). In this context, it is important to highlight that we assessed the exposure of TCS and DEHP in HepG2 cells, which although a tumoral cell line, they express phases 1 and 2 metabolizing enzymes (Mersch-Sundermann et al., 2004), which may influence to toxicity related to the TCS and DEHP exposure.

Moreover, it is well known that laboratory model experiments are run under restricted controlled conditions; once in the environment, studying the interactions between these toxicants is more complex, since they can interact with more than one compound and be metabolized by bacteria, or suffering alterations by UV radiation from sunlight, for example (Rehberger et al., 2018). For this reason, studies involving mixtures of toxicants are important to emphasize the risk of these compounds to the environment and human health and it can contribute with primary data for assessing exposure biomarkers related to DNA damage and further studies with other compounds in other cells models or in *in vivo* models.

Taken together, our results provide further evidence concerning the mutagenic effects of TCS and DEHP in mammalian cells. Also, DNA damage induced by exposure to the both compounds may be related to delay in cell cycle and to acute toxicity. Interestingly, the most significant damages were related to exposure of lower doses of TCS, DEHP, as well as their combinations, showing that EDCs are able to induce several disturbances on cells, even at low concentrations.

## Data Availability Statement

The raw data supporting the conclusions of this article will be made available by the authors, without undue reservation.

## Author Contributions

GB, LL, and ND: conceptualization. EN, FM, LL, and ND: methodology, investigation. MK: statistics. EN, GB, and ND: writing original draft preparation. GB and ND: writing, review, and editing. All authors contributed to the article and approved the submitted version.

## Conflict of Interest

The authors declare that the research was conducted in the absence of any commercial or financial relationships that could be construed as a potential conflict of interest.
