# Supplementary material for: Acute Toxicity and DNA Instability Induced by Exposure to Low Doses of Triclosan and Phthalate DEHP, and Their Combinations, in vitro
Source: Front Genet. 2021 Apr 20;12:649845. doi: 10.3389/fgene.2021.649845 (PMC8093768; doi:10.3389/fgene.2021.649845)
Supplement: Supplementary file 1 [file Image_1.pdf]

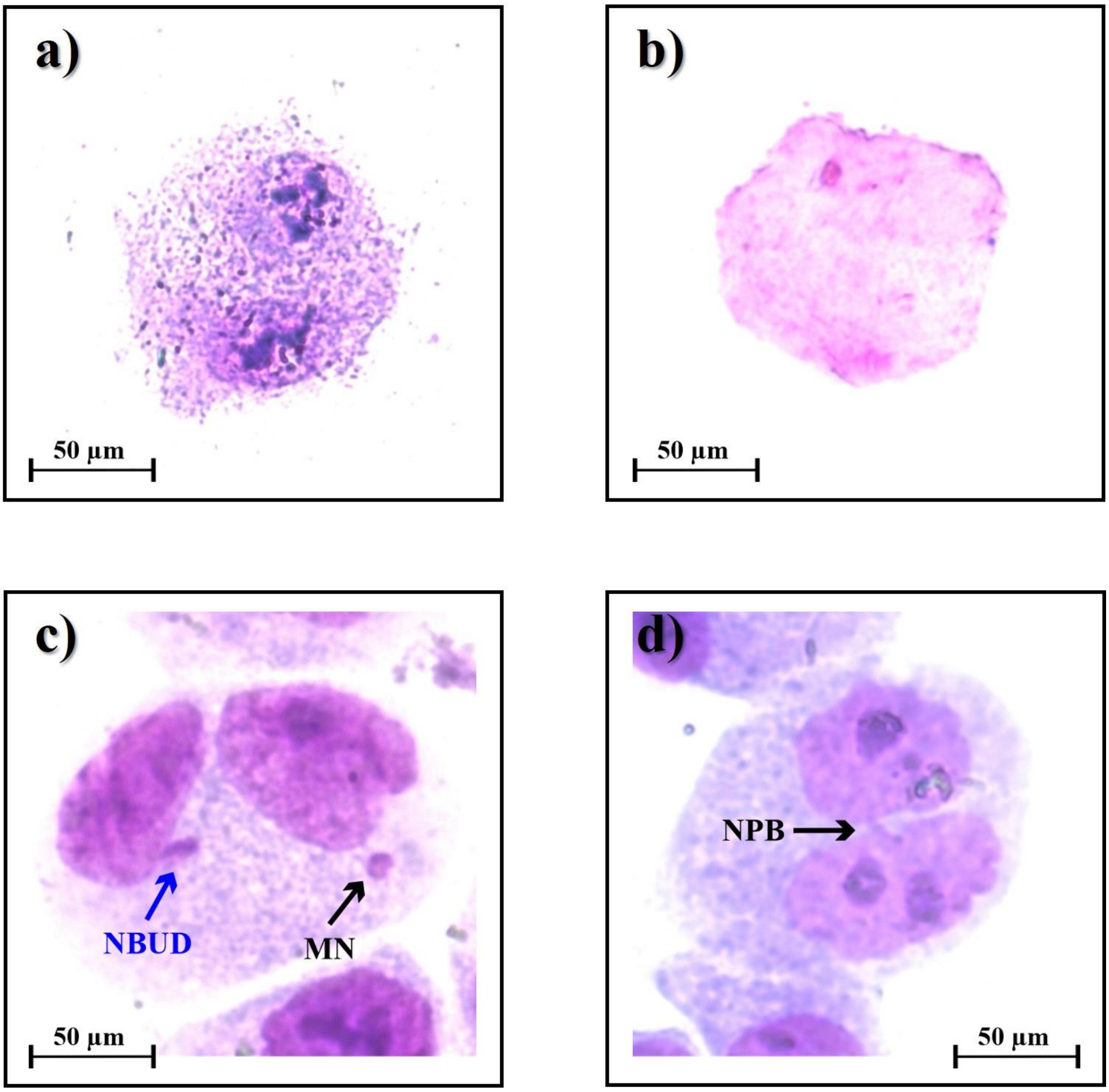

Figure S1: Biomarkers assessed by the cytokinesis-block micronucleus cytome (CBMNCyt) assay in HepG2 cells: a) apoptotic cell treated with TCS 1.0 µM; b) necrotic cell treated with TCS 1.0 µM; c) binucleated cell treated with TCS 1.0 µM + DEHP 1.0 µM showing nuclear abnormalities: nuclear bud (NBUD, blue arrow) and micronucleus (MN, black arrow); and d) binucleated cell treated with DEHP 1.0 µM showing nucleoplasmatic bridge (NPB; arrow). All pictures were taken by light microscopy at magnification of 630x (AxioLab A1, Carl Zeiss, Jena, Germany).
